# Laceration of the Cervix Uteri

**Published:** 1879-09

**Authors:** 


LACERATION OF CERVIX UTERI. — Dr. S. C. Thompson, of Cedarville, Illinois, sends us an account of a case he regards as one of laceration of the cervix uteri, giving rise to severe and protracted suffering; and which was ultimately cured by wearing a well adjusted Fowler's pessary. Hence he asks the question, whether, the proper and skillful use of this particular pessary may not be substituted for Emmet's operation, in the treatment of this injury.

